# Measuring Surgical Waiting Times in Breast Cancer: Admission to Surgery Versus Biopsy Result to Surgery

**DOI:** 10.3390/healthcare13233010

**Published:** 2025-11-21

**Authors:** Cem Tandoğan, Mustafa Berkeşoğlu, Ferah Tuncel, Didem Derici Yıldırım, Cumhur Özcan, Sami Benli, Erkan Güler, Eda Bengi Yılmaz

**Affiliations:** 1 Department of General Surgery, Dörtyol State Hospital, 31600 Dörtyol, Hatay, Türkiye; 2 Department of General Surgery, Faculty of Medicine, Mersin University, 33110 Mersin, Türkiye; 3 Department of Pathology, Faculty of Medicine, Mersin University, 33110 Mersin, Türkiye; 4 Department of Biostatistics and Medical Informatics, Faculty of Medicine, Mersin University, 33110 Mersin, Türkiye; 5 Department of Radiation Oncology, Faculty of Medicine, Mersin University, 33110 Mersin, Türkiye

**Keywords:** breast neoplasms, critical pathways, quality indicators, health care, time-to-treatment, waiting lists

## Abstract

**Background:** Preoperative timelines may lengthen due to tailored evaluation and system constraints. We examined whether two complementary measures of time-to-surgery (TTS)—admission-to-surgery (A-TTS) and biopsy-result-to-surgery (B-TTS)—behave similarly and whether parallel tracking offers service value. **Methods:** In a single-center retrospective cohort of eligible women undergoing upfront surgery for invasive breast cancer (2010–2021; *n* = 167), we reported quality indicators for timeliness (target attainment, agreement and discordance, the interval gap, and the surgery-to-adjuvant interval), while analyzing recurrence as the primary endpoint and overall survival as secondary. Discrimination analyses, logistic regression, and Cox models were used; non-proportional hazards were handled with a log–time interaction centered at 24 months. **Results:** The two time measures were not interchangeable: discordant cases were frequent and pointed to different bottlenecks. A-TTS ≤ 24 days was independently associated with recurrence (OR 3.16; 95% CI 1.13–8.82) and showed a large early hazard for death at 24 months that attenuated over time (HR 22.83; 95% CI 6.44–80.98; interaction HR 0.06; 95% CI 0.02–0.21), whereas B-TTS showed no association. **Conclusions:** Lymphovascular invasion remained the strongest pathologic correlate of survival. Tracking both intervals, paired with brief, reason-coded reviews of discordant cases, may support scheduling, quality dashboards, and breach governance better than a single TTS metric.

## 1. Introduction

Breast cancer is biologically diverse, yet often curable when care is coordinated across surgery, systemic therapy, and radiotherapy (RT) within multidisciplinary teams (MDTs) that integrate diagnostic imaging and pathology into preoperative planning [1,2].

The preoperative interval can lengthen due to tailored evaluation (e.g., axillary ultrasonography [US] for nodal assessment with selective magnetic resonance imaging [MRI], genetic counseling/testing, reconstruction planning, and optimization of comorbidities) and system constraints (operating room capacity, staffing, and inter-institutional transitions), as MDTs triage upfront surgery versus neoadjuvant systemic therapy (NAST) [1,2,3,4,5,6,7]. Such tailored work-ups may legitimately extend pathways but can be perceived as risky delays, potentially heightening patient anxiety (“waiting = spread”) and inviting medicolegal scrutiny.

Determinants of timing are not interchangeable: very short in-hospital intervals may signal prioritization, whereas longer diagnosis-to-surgery intervals can reflect planning needs or system strain and do not necessarily indicate poorer care [4,5,7,8]. Although several cohorts and meta-analyses associate longer waits with worse survival, with inflections around 8–9 weeks and subtype-specific vulnerability, these associations may reflect selection and system effects rather than causality [9,10,11,12]. Very short admission-to-surgery (A-TTS) intervals can also indicate urgency rather than benefit, consistent with the waiting-time paradox in symptomatic cohorts [13].

To align measurement with scheduling decisions and governance, we distinguished time-to-surgery (TTS) from time-to-treatment-initiation (TTI) and prespecified two complementary intervals—biopsy-result-to-surgery (B-TTS) and A-TTS—designating A-TTS as the primary measure. We then examined their relationships with recurrence and overall survival (OS) to inform counseling, scheduling, and quality improvement.

## 2. Materials and Methods

### 2.1. Study Design, Setting, and Participants

**Figure 1:**
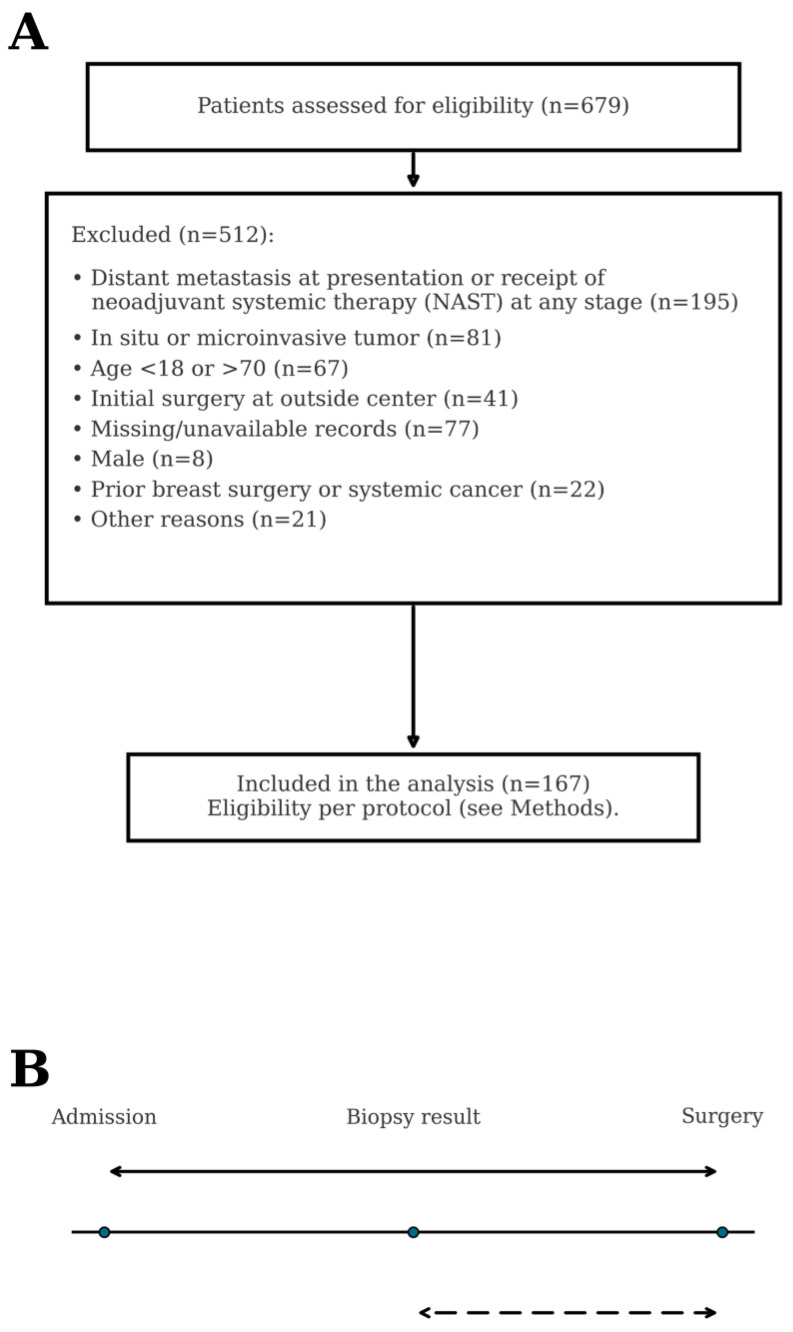
(**A**) Participant flow: screening, prespecified exclusions, and the final analytic cohort (n = 167). (**B**) Care-pathway schematic: A-TTS (solid arrow) and B-TTS (dashed arrow).

This retrospective, single-center cohort study was conducted at Mersin University Faculty of Medicine Hospital, a tertiary-care academic center, with accrual from 1 January 2010 to 31 December 2021. Reporting followed the Strengthening the Reporting of Observational Studies in Epidemiology (STROBE) guidelines [14]. The study was approved by the Mersin University Clinical Research Ethics Committee (approval no. 2022/18/656). The requirement for informed consent was waived due to the retrospective, de-identified design, in line with national regulations and the Declaration of Helsinki. The screening and selection process is shown in Figure 1A; 167 consecutive patients comprised the final analytic cohort.

Eligible patients were women aged 18–70 years who underwent definitive surgery for invasive breast carcinoma at our institution with complete diagnostic, treatment, and follow-up data; no distant metastasis at presentation; and no receipt of NAST. Exclusion criteria were distant metastasis at presentation or receipt of NAST at any stage; in situ or microinvasive disease only (pT1mi); age < 18 or >70 years; initial definitive surgery performed at another center; missing/unavailable records (lacking essential information for endpoint derivation—no institutional admission/scheduling entry for A-TTS, no verifiable signed report date for the index biopsy required to derive B-TTS, or incomplete key baseline data); male sex; prior breast surgery; history of another invasive malignancy; and other prespecified reasons (withdrawal of/deferral of definitive surgery, transfer of care to another facility before surgery, incidental malignancy outside the index episode) (Figure 1A). Imaging completeness was not an inclusion criterion because these variables were not required to derive primary timing metrics (all index core biopsies were US-guided; mammography [MMG]/MRI were recorded when available). Patients presenting with an external index diagnostic core biopsy were excluded a priori from all analyses to avoid pre-admission bias in TTS metrics; admission and biopsy-result timestamps were, therefore, not derived for external referrals. Core timing variables (A-TTS, B-TTS) and outcomes (recurrence, vital status) were complete; a few non-core covariates had sporadic missingness and were analyzed with available-case denominators. The analytic dataset comprised 167 consecutive women undergoing upfront surgery, with 18 recurrences and 23 deaths over follow-up; class distributions for baseline variables are summarized in Table 1 and Supplementary Tables S1–S6, and the cohort assembly is shown in Figure 1A.

### 2.2. Data Sources and Variables

Data were abstracted from the institutional electronic health record, operative notes, radiology reports (US; MMG and breast MRI as recorded), and final pathology reports.

Staging adhered to the American Joint Committee on Cancer (AJCC) 8th edition. For cases diagnosed before 1 January 2018, restaging was performed retrospectively using source pathology data to ensure uniformity across the cohort. We used anatomic stage groups (I–III) and analyzed N category (N0–N3) as an ordinal per-category variable. Histology was grouped into three categories: (i) no special type (NST) and mixed/unfavorable variants (NST, mixed, micropapillary, metaplastic, pleomorphic invasive lobular carcinoma [ILC]); (ii) classic ILC; and (iii) favorable special types (mucinous, papillary, cribriform, tubular), applying a ≥90% single-pattern rule. Mammographic density was abstracted as recorded: fatty, scattered, heterogeneously dense, and extremely dense.

Breast procedure was recorded as breast-conserving surgery (BCS) or mastectomy (simple mastectomy or mastectomy with immediate implant-based reconstruction); axillary procedure was recorded as sentinel lymph node biopsy (SLNB) and/or axillary lymph node dissection (ALND). The receipt of adjuvant chemotherapy, RT, endocrine therapy, and human epidermal growth factor receptor 2 (HER2)-targeted therapy (when indicated) was coded as binary (yes/no).

US-measured tumor size at diagnostic biopsy and pathologic invasive tumor size on the surgical specimen were abstracted in millimeters (mm). Pathologic size was categorized as ≤20 mm, >20–50 mm, and >50 mm. The presence of ductal carcinoma in situ (DCIS) accompanying invasive carcinoma was recorded. Intrinsic subtype (immunohistochemical [IHC] surrogate) was classified as Luminal A (estrogen receptor [ER] and/or progesterone receptor [PR] positive, HER2 negative), Luminal B (ER and/or PR positive with variable HER2 status), HER2-enriched (ER/PR negative, HER2 positive), or triple-negative (ER/PR/HER2 negative). Lymphovascular invasion (LVI) and perineural invasion (PNI) were recorded per standard histopathology, and Nottingham grade (I–III) assigned. Multifocality (≥2 invasive foci within the same quadrant) and multicentricity (foci in different quadrants or >5 cm apart within the same breast) were coded as present/absent (yes/no). Margin status followed contemporary evidence-based guidelines: negative, no ink on invasive carcinoma; positive, ink on tumor. Where an in situ component lay ≤1 mm from the inked edge without ink on invasive carcinoma, margins were described as “close” and analyzed as negative. Pure DCIS was absent; therefore, the 2 mm DCIS threshold did not apply [15,16,17].

### 2.3. Diagnostic-to-Treatment Intervals

Admission was defined as the date of first registration/initial clinical contact for the index breast cancer at our institution; preoperative assessments were predominantly outpatient. Surgery date corresponded to the date of first definitive breast cancer surgery. A-TTS was defined as the admission-to-surgery interval; B-TTS as the biopsy-result-to-surgery interval (Figure 1B). All intervals were recorded in days. From A-TTS and B-TTS, we also derived six indicators for service monitoring: (i) attainment of A-TTS ≤ 24 days; (ii) attainment of B-TTS ≤ 24 days; (iii) agreement statistics (overall agreement, Cohen’s kappa (κ) with 95% confidence intervals (CIs) estimated by bias-corrected and accelerated bootstrap with 5000 resamples, positive and negative percentage agreement) and the exact McNemar test; (iv) discordant cases (B-TTS ≤ 24 with A-TTS >24); (v) median A–B gap (A-TTS minus B-TTS); and (vi) median surgery-to-adjuvant interval. These indicators were defined a priori for service/quality monitoring rather than biological inference. A-TTS served as the primary timing construct. Its operating threshold was derived by receiver-operating characteristic (ROC) analysis using Youden’s J and then applied in selected categorical analyses; the same ≤24-day indicator was used for B-TTS to enable direct agreement testing. As a robustness benchmark, we also evaluated a pragmatic 30-day operational benchmark (~1 month) used locally for capacity planning. B-TTS was summarized descriptively and examined for concordance with A-TTS. Logistic regression and a decision tree were compared using paired procedures on the same patient-level predictions: DeLong’s test for ΔAUC; McNemar’s test at (i) model-specific Youden’s J cut-points and (ii) a prevalence-matched threshold; and 2000× bootstrap 95% confidence intervals for paired differences in accuracy, F1-score, positive predictive value (PPV), and negative predictive value (NPV). The decision tree was depth-limited and pruned a priori to reduce variance and maintain interpretability. All tests were two-sided (α = 0.05). Given the small event count, inferences were interpreted with caution. The admission date was the earliest institution-side registration that opened the index episode (first on-site clinic visit or a pre-visit triage/registration entry). The biopsy-result date was the verifiable signed date on the diagnostic histopathology report that first established invasive carcinoma; specimen-collection dates and undated/unsigned external documents did not qualify.

### 2.4. Outcomes and Follow-Up

The primary outcome was breast cancer recurrence (any locoregional or distant relapse) over follow-up. The secondary outcome was OS, defined as time from surgery to death from any cause; patients alive at last contact were censored. Time-to-event and follow-up were measured in months; potential follow-up was summarized by reverse Kaplan–Meier. Deaths were ascertained from the electronic health record and institutional mortality notifications. Recurrence time was defined from the date of surgery to the first documented locoregional or distant recurrence; patients without recurrence were censored at last contact or death.

### 2.5. Statistical Analysis

All tests were two-sided (α = 0.05). Continuous variables were summarized as mean ± SD when approximately normal and otherwise as median (IQR); between-group comparisons used the *t*-test or Mann–Whitney U test, as appropriate. Normality was assessed by visual inspection (histograms/Q–Q plots) and the Shapiro–Wilk test. Categorical associations used Pearson’s χ^2^ or Fisher’s exact test. Contingency-table percentages were reported as column percentages. No imputation or additional normalization/encoding was performed; continuous variables were analyzed in their native units (with A-TTS/B-TTS also expressed per 10-day increments) and categorical predictors were coded as factors. To address prioritization bias, unadjusted comparisons were complemented by stratified agreement summaries (by pathologic stage and nodal status) and by adjusted analyses (logistic for recurrence; Cox for overall survival) controlling for prespecified baseline prognostic factors (age [years], pathologic stage [I–II vs. III], nodal status [negative vs. positive], tumor size [mm], and LVI). Given the small numbers of events (recurrences and deaths), models were kept parsimonious and no automated feature-selection was used. Clinically relevant covariates were prespecified, and redundancy/collinearity was screened a priori using pairwise associations (Cramér’s V for categorical pairs; Spearman’s ρ for continuous pairs) and variance-inflation factors; only one encoding per construct (e.g., stage, size, A-TTS threshold) was entered in any given model (Supplementary Tables S1 and S2).

#### 2.5.1. Agreement Between Timing Definitions

To compare classifications based on A-TTS and B-TTS, we computed overall agreement; Cohen’s κ with 95% CI estimated by bias-corrected and accelerated (BCa) bootstrap, 5000 resamples; positive and negative percentage agreement; and the exact McNemar test of marginal homogeneity. As a sensitivity analysis, metrics were re-estimated at a pragmatic 30-day threshold (Supplementary Table S3). Supplementary Figures S1 and S2 show additional diagnostics.

#### 2.5.2. Recurrence Modeling

Recurrence was modeled with binary logistic regression. A-TTS (continuous and dichotomized at the ROC-derived cut-off) and clinically defined covariates were considered; closely related variables were not modeled together to avoid redundancy/collinearity, given the limited number of events. Collinearity was screened using pairwise associations (phi/Cramér’s V for nominal pairs; Spearman rank correlations for ordinal/continuous mixes) and by inspecting tolerance and variance inflation factors from an ordinary least squares regression including candidate predictors. Adjuvant therapies (chemotherapy, RT, endocrine therapy) were not included as predictors to avoid confounding by indication and time-dependent bias. Model performance was summarized by area under the ROC curve, Hosmer–Lemeshow goodness-of-fit, Brier score, and Nagelkerke R^2^. Calibration was assessed with bootstrap-based curves (apparent vs. bias-corrected; 200 replicates). As a small-sample sensitivity, we also fitted an exact (conditional) logistic regression, reporting exact *p*-values and 95% confidence intervals. To address potential prioritization (triage) bias without invoking an arbitrary day cut-off, we used propensity-score (PS) adjustment and robustness checks: PS common-support trimming (0.10–0.90), exclusion of the fastest scheduling decile (lowest 10% of A-TTS), and advanced-disease exclusions (stage III or >50 mm or N2–3). The PS for A-TTS ≤ 24 was estimated by logistic regression using the prespecified baseline factors (age, pathologic stage I–II vs. III, nodal status, tumor size in mm, and LVI) and applied both as a continuous covariate and in quintiles. In sensitivity analyses, A-TTS and B-TTS were entered as continuous predictors (per 10 days); because prioritization bias is linked to A-TTS logistics, the PS was constructed for A-TTS ≤ 24, while B-TTS was assessed via agreement metrics and continuous sensitivity models. Thresholded classification was summarized at the Youden’s J cut-point on the model-predicted probability and at a prevalence-matched probability threshold targeting the observed prevalence (≈0.108; 18/167); corresponding confusion matrices for both thresholds were provided (Supplementary Table S4). In sensitivity analyses, recurrence was also modeled using a cause-specific Cox model with time origin at surgery and endpoint at first recurrence. Deaths without prior recurrence were censored. Proportional hazards were assessed with Schoenfeld residuals, and hazard ratios with 95% confidence intervals were reported.

#### 2.5.3. Overall Survival

Deaths without prior recurrence were censored in a cause-specific framework, and patients alive at last contact were administratively censored. OS was analyzed with Cox regression (ties handled by the Efron method). Proportional hazards were evaluated using Schoenfeld residuals (global and covariate-specific tests). When the proportional-hazards assumption was violated for A-TTS ≤ 24 days, we specified an extended Cox model with a log–time interaction using ln(time/24), where time is in months; the main effect is the hazard ratio at 24 months, and an interaction hazard ratio <1 indicates attenuation over time. Where applicable, bootstrap resampling (2000 samples) was used to obtain robust 95% confidence intervals. For OS, A-TTS and B-TTS were likewise evaluated per 10 days in Cox models. OS models followed the same prespecification and redundancy screening strategy (no automated feature selection/importance; pairwise associations and tolerance/VIF to limit collinearity).

#### 2.5.4. Exploratory Subgroups

A-TTS (dichotomized at a data-driven ROC cut-off) was assessed within intrinsic subtype and HER2 status (positive vs. negative). For recurrence, Fisher’s exact tests and ORs with 95% CIs; for OS, Kaplan–Meier curves were compared using the log-rank test. Effect modification was examined by adding interaction terms (A-TTS × intrinsic subtype; A-TTS × HER2 status) to the logistic and Cox models and evaluating Wald tests. Exploratory analyses were not adjusted for multiplicity.

#### 2.5.5. Software and Presentation

All analyses (descriptive summaries, group comparisons, ROC, logistic regression, Kaplan–Meier and Cox models—including log–time interactions) were performed in R, version 4.5.1 (R Foundation for Statistical Computing, Vienna, Austria). Figures were generated from R output and redrawn for stylistic consistency and resolution. Diagnostic-to-treatment intervals are reported in days; time-to-event outcomes and follow-up in months. All point estimates (κ, AUC, OR/HR) are reported with 95% confidence intervals.

## 3. Results

### 3.1. Cohort and Outcomes

Among 167 patients, node-positive disease was present in 58.7%, grade III in 49.7%, and anatomic stage II–III in 75.4%; LVI and PNI were observed in 56.3% and 37.1%, respectively. Most patients underwent mastectomy (85.0%) and received adjuvant systemic therapy (90.4%). Over follow-up, 18 recurrences (10.8%) and 23 deaths (13.8%) occurred. Median potential follow-up was 61.0 months with reverse Kaplan–Meier; median follow-up was 52.0 months (IQR 33.5–72.0). Histology was predominantly NST/mixed (82.0%); in situ components accompanied invasion in 68.3%; multifocal/multicentric disease was 21.0% (Table 1).

In the overall cohort (*n* = 167), median (IQR) values were biopsy tumor size 20.0 mm (14.0–26.0), pathologic invasive tumor size 25.0 mm (19.5–40.0), Ki-67 15.0% (10.0–30.0), A-TTS 35.0 days (25.0–51.5), B-TTS 16.0 days (9.0–25.0), A–B 15.0 days (10.0–24.0), surgery-to-adjuvant interval 43.0 days (33.5–55.5), survival time 58.0 months (38.0–110.0), and follow-up time 52.0 months (33.5–72.0). Mammographic density (available for 80.2% of patients) was not associated with recurrence.

Patterns of recurrence (*n* = 18) were predominantly distant (15/18): bone (*n* = 7), lung (*n* = 6), liver (*n* = 1), and contralateral axillary lymph nodes (*n* = 1; classified as distant per AJCC 8th); locoregional events included ipsilateral axillary nodes (*n* = 2) and ipsilateral breast (*n* = 1). Among patients with recurrence, 11/18 (61.1%) died during follow-up.

Eight cases had positive or close margins; in invasive ± DCIS, margin status was adjudicated by the invasive component [15]. One patient had a positive posterior (deep) margin on the pectoral fascia; no re-excision was performed, and the patient remained recurrence-free at 58 months following adjuvant RT and systemic therapy.

### 3.2. Diagnostic-to-Treatment Intervals

A-TTS exceeded B-TTS (medians 35 vs. 16 days); the within-patient difference (A–B) had a median of 15 days (IQR 10–24). Using the ROC-derived 24-day operating threshold and a pragmatic 30-day benchmark, A-TTS met the target less often than B-TTS (24.6% vs. 73.7% at 24 days; 40.1% vs. 80.2% at 30 days) (Table 1 and Table 2; Supplementary Figure S3). At 24 days, overall agreement was 50.9% with fair concordance (Cohen’s κ = 0.21; 95% CI 0.14–0.29) and marked asymmetry—82 patients met B-TTS ≤ 24 but not A-TTS ≤ 24, whereas no patient met A-TTS ≤ 24 but not B-TTS ≤ 24 (exact McNemar *p* < 0.001)—indicating limited interchangeability. At 30 days, agreement increased to 59.9% (Cohen’s κ = 0.28; 95% CI 0.19–0.38), but asymmetry persisted—67 patients met B-TTS ≤ 30 but not A-TTS ≤ 30, whereas no patient met A-TTS ≤ 30 but not B-TTS ≤ 30 (*p* < 0.001) (Supplementary Table S3). In severity-adjusted models, A-TTS ≤ 24 was associated with recurrence (logistic OR 2.86, 95% CI 0.97–8.41, *p* = 0.056) and overall survival (Cox HR 1.65, 95% CI 0.67–4.04, *p* = 0.278); using a 30-day cut-off yielded OR 1.76 (95% CI 0.62–4.98, *p* = 0.285) and HR 2.38 (95% CI 0.996–5.71, *p* = 0.051). In an ancillary model with A-TTS ≤ 24 as the outcome, stage, nodal status, LVI, tumor size (per mm), and age did not independently predict earlier surgery (e.g., stage III vs. I–II OR 0.29, 95% CI 0.06–1.40; *p* = 0.123). For A-TTS, AUC = 0.672 (95% CI 0.538–0.807; *p* = 0.017); at 24 days, sensitivity was 50.0% and specificity 78.5%. In a service-quality framing, 49.1% met B-TTS but not A-TTS (discordant profile), and the surgery-to-adjuvant interval had a median of 43 days. In PS-adjusted models, A-TTS ≤ 24 was not associated with recurrence (OR 1.65, 95% CI 0.26–10.53, *p* = 0.595 with PS quintiles; OR 1.58, 95% CI 0.26–9.83, *p* = 0.622 with continuous PS) or overall survival (HR 2.63, 95% CI 0.43–16.10, *p* = 0.296). In the same PS-adjusted Cox model, LVI remained independently associated with worse OS (HR 6.33, 95% CI 1.14–35.04; *p* = 0.035). Excluding the fastest scheduling decile (lowest A-TTS decile) yielded directionally consistent, non-significant estimates for recurrence (OR 2.94, 95% CI 0.44–19.38, *p* = 0.264) and survival (HR 2.25, 95% CI 0.42–12.07, *p* = 0.344). Results were similar after PS common-support trimming (0.10–0.90) and after advanced-disease exclusions. In sensitivity analyses treating the intervals as continuous exposures (per 10 days), A-TTS showed no statistically significant association with recurrence (OR 0.73, 95% CI 0.53–1.01; *p* = 0.058) or overall survival (HR 0.94, 95% CI 0.76–1.15; *p* = 0.534), with a similar pattern for B-TTS (recurrence OR 0.85, 95% CI 0.58–1.24; *p* = 0.391; OS HR 0.95, 95% CI 0.70–1.28; *p* = 0.718); in a PS-adjusted Cox model including LVI and the A-TTS propensity score, the per-10-day effect of A-TTS remained non-significant (HR 0.98, 95% CI 0.77–1.25; *p* = 0.866), while LVI was borderline (HR 3.61, 95% CI 0.98–13.34; *p* = 0.054). These continuous results were directionally consistent with the categorical analyses and did not alter conclusions. Paired testing showed no significant difference in discrimination between logistic regression and decision tree (ΔAUC = −0.024; 95% CI −0.096 to 0.047; *p* = 0.506), and classification performance was also similar at the prevalence-matched threshold (McNemar *p* = 0.383; Supplementary Table S5).

### 3.3. Recurrence

On univariable analyses, recurrence was associated with higher pathologic N category, stage III (vs. I–II), LVI, PNI, larger tumor size (continuous and >50 mm), and shorter A-TTS with a smaller A–B interval; B-TTS showed no association (Table 2; Supplementary Table S6). In a parsimonious logistic model including A-TTS ≤ 24 days, nodal positivity, and LVI, A-TTS ≤ 24 days remained independently associated with recurrence (OR 3.16; 95% CI 1.13–8.82; *p* = 0.028). Discrimination was moderate (AUC 0.733; 95% CI 0.620–0.847) (Table 3). Threshold-specific operating characteristics and confusion matrices at the Youden’s J and prevalence-matched thresholds (target prevalence ≈ 0.108 [18/167]; achieved ≈ 0.150 [25/167]) are reported in Supplementary Table S4. Adjusted ORs are shown in Supplementary Figure S4; ROC and calibration plots are shown in Supplementary Figures S1 and S2. The cause-specific Cox analysis was directionally consistent with the logistic model, and no proportional-hazards violations were detected on Schoenfeld diagnostics. In an exact (conditional) logistic sensitivity analysis, A-TTS ≤ 24 remained associated with recurrence (OR 3.16; 95% CI 1.13–8.82; exact *p* = 0.028), with effect direction unchanged relative to the primary model. At a 30-day benchmark, A-TTS ≤ 30 was not statistically significant (OR 1.93; 95% CI 0.71–5.30; *p* = 0.200); inferences were unchanged.

### 3.4. Overall Survival

**Figure 2:**
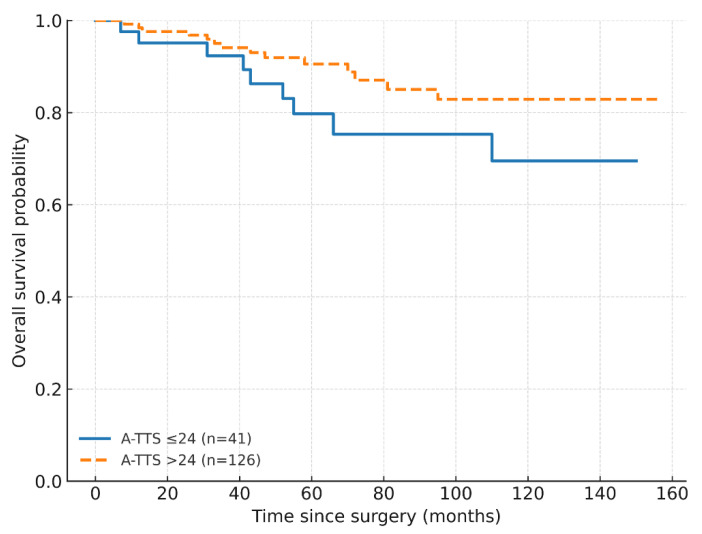
Overall survival by A-TTS ≤ 24 days versus >24 days (Kaplan–Meier). Numbers at risk are shown below the *x*-axis. Abbreviations: A-TTS, admission-to-surgery.

**Figure 3:**
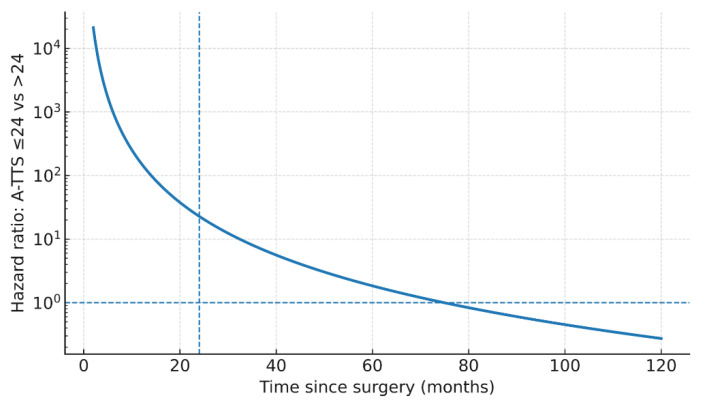
Time-varying effect of A-TTS ≤ 24 days on overall survival in the extended Cox model: hazard ratio at 24 months and log–time interaction ln(time/24) with 95% confidence bands. Abbreviations: A-TTS, admission-to-surgery.

In Cox proportional-hazards models (Efron ties), LVI was associated with worse OS (HR 3.93; 95% CI 1.03–14.99; *p* = 0.045), whereas A-TTS ≤ 24 and nodal status were not statistically significant (A-TTS ≤ 24: HR 1.73; 95% CI 0.75–4.02; *p* = 0.201); nodal: HR 1.23; 95% CI 0.36–4.21; *p* = 0.737). At a 30-day benchmark, A-TTS ≤ 30 showed a non-significant trend (HR 2.23, 95% CI 0.96–5.18; *p* = 0.062); LVI was borderline (HR 3.71, 95% CI 0.95–14.40; *p* = 0.059), and nodal status remained non-significant. In extended Cox models with a log–time interaction ln(time/24), the HR at 24 months for A-TTS ≤ 24 days was 22.83 (95% CI 6.44–80.98; *p* < 0.001) and attenuated over time (interaction HR 0.06; 95% CI 0.02–0.21; *p* < 0.001) (Table 4; Figure 2 and Figure 3; Supplementary Figure S5).

### 3.5. Exploratory Analyses

The association of A-TTS ≤ 24 days with recurrence appeared to cluster in hormone receptor-positive and in HER2-negative disease (for HER2-negative: 8/33 vs. 8/105; OR 3.88; 95% CI 1.33–11.36; *p* = 0.024), with no clear association in HER2-enriched or triple-negative subtypes. For OS, a nominal difference was seen only in the HER2-negative stratum (log-rank *p* = 0.031). Formal interaction tests were non-significant (A-TTS × intrinsic subtype, *p* = 0.397; A-TTS × HER2 status, *p* = 0.850). Subgroup analyses were exploratory and not adjusted for multiplicity.

## 4. Discussion

In our cohort, A-TTS ≤ 24 days was associated with higher recurrence risk and an early, attenuating mortality hazard, whereas B-TTS showed no association with either endpoint. LVI appeared to be the strongest pathologic correlate of OS. Agreement between the two timing measures was only fair (κ = 0.21 [95% CI 0.14–0.29] at 24 days; κ = 0.28 [95% CI 0.19–0.38] at 30 days), and A-TTS classified fewer patients as meeting time targets than B-TTS (medians 35 vs. 16 days). This discordance suggests that A-TTS and B-TTS are not interchangeable and may warrant parallel tracking for quality improvement [18]. The early, waning hazard in the extended Cox model is compatible with prioritization bias (confounding by indication) as a working explanation. We therefore analyzed them separately to align measurement with distinct segments of care. After adjustment for age, pathological stage (I–II vs. III), nodal status (negative vs. positive), tumor size (per mm), and LVI, A-TTS ≤ 24 was not associated with lower risks of recurrence or overall survival. Earlier scheduling (A-TTS ≤ 24) was also not independently predicted by higher stage, nodal status, tumor size, or age, which supports prioritization/indication bias, rather than a direct causal effect of shorter A-TTS. Findings were directionally consistent when a month-based 30-day benchmark was applied. Accordingly, we interpret A-TTS and B-TTS as workflow/process indicators, rather than causal determinants of prognosis.

Across contemporary cohorts and meta-analyses, longer TTS correlates with worse survival, with risk rising beyond approximately 8–9 weeks; operational thresholds vary by case-mix and system factors [10,11,19,20,21]. Intrinsic subtype may also modify delay-associated mortality [12], and health-system reports describe TTS increases independent of tumor biology [22]. Against this backdrop, consensus statements and guidelines acknowledge individualized diagnostic work-up and reconstructive planning as legitimate drivers of longer pathways, while cautioning against one-size-fits-all time targets [1,2,23,24,25].

The paradoxical association of shorter A-TTS with worse outcomes is consistent with prioritization bias, whereby higher-acuity cases are triaged more rapidly from admission to surgery [26]. Contemporary United Kingdom (UK) policy and monitoring frameworks recognize that clinically appropriate care may breach time standards and should be interpreted in context [27,28,29]. As neoadjuvant indications broaden, the preoperative work-up may legitimately extend. Although we did not measure waiting-related anxiety or medicolegal context, the lack of an association for B-TTS and the early, waning A-TTS signal may support interpreting context-driven extensions as clinically appropriate, rather than biologically harmful. Accordingly, timing metrics may be interpreted alongside clinical circumstances rather than against rigid thresholds. In PS-adjusted analyses addressing potential triage bias, A-TTS ≤ 24 did not show a statistically significant association with OS, whereas LVI remained the most consistent pathologic correlate.

Two timing constructs are preferable to one because they capture different segments of the preoperative pathway. A-TTS summarizes the interval from first registration or initial clinical contact to surgery and is sensitive to institutional throughput and clinical prioritization, whereas B-TTS anchors timing to the biopsy result and is more sensitive to diagnostic work-up and planning. Relying on a single, undifferentiated TTS measure risks misclassification. Tracking both measures in parallel and auditing discordant cases can localize bottlenecks without implying biological harm: when B-TTS meets its target but A-TTS does not, delays likely occurred before the result (imaging or biopsy scheduling, pathology turnaround time, integration of external reports); the reverse pattern points to delays after the result (MDT decisions, reconstructive planning, preoperative anesthesia assessment, operating room scheduling). Brief, reason-coded documentation of timing decisions and simple process mapping align with quality frameworks and waiting-time standards and can reduce patient anxiety while providing governance/medicolegal clarity when individualized evaluation appropriately extends timelines. Accordingly, we summarize six process-quality indicators to operationalize parallel monitoring and brief, reason-coded breach reviews [1,2,21,27,28,29].

Exploratory subgroup signals—more apparent in hormone receptor-positive and HER2-negative disease, with no consistent pattern in HER2-enriched or triple-negative subtypes—should be interpreted cautiously. These observations are hypothesis-generating; interaction tests were non-significant and power was limited, warranting external validation [12].

Across models, LVI appeared to be the most consistent pathologic predictor for OS, aligning with evidence that lymphovascular permeation reflects aggressive biology independent of diagnostic-to-treatment timing [30,31,32]. Taken together, TTS metrics may be more appropriately viewed as process indicators, whereas tumor biology may provide a more stable prognostic signal.

This single-center retrospective design limits causal inference; unmeasured triage factors, capacity constraints, high-risk case mix, and limited events may confound associations. The ROC-derived A-TTS cut-off may be optimistic and requires external validation. Although waiting-related anxiety and the medicolegal context were not measured, context-aware use of A-TTS/B-TTS with brief, reason-coded reviews of threshold breaches may indirectly alleviate patient anxiety and strengthen medicolegal clarity; overall, findings are hypothesis-generating and operationally exploratory and warrant prospective evaluation. Given that the limited events-per-parameter (EPV ≈ 3–4) may limit precision; our prespecified parsimony mitigates overfitting, and penalized approaches (e.g., ridge/LASSO/Firth) can be explored in future validation. Models were kept interpretable—logistic regression provides coefficient-level transparency (ORs with 95% CIs), and the decision tree was intentionally shallow/pruned to preserve rule readability. Strengths include a consecutive, well-defined cohort; explicit separation of two complementary timing constructs (A-TTS and B-TTS) with harmonized definitions; parsimonious modeling that respects EPV constraints; formal checks for non-proportional hazards via log–time interactions; and agreement analyses (κ, McNemar) that clarify why a single undifferentiated TTS metric can mislead—together yielding an interpretable, operationally relevant framework for quality improvement. Generalizability may vary because resource constraints and scheduling policies at our tertiary center (e.g., operating-room capacity, reconstructive planning queues, pathology turnaround) may differ from other systems. Future external validation should include subgroup calibration/fairness checks across key strata (e.g., age, stage, intrinsic subtype) to assess demographic bias and transportability. Therefore, thresholds and breach-review workflows should be calibrated to local service models, rather than assumed to reflect universal biological risk. Low EPV and single-center case mix may foster overfitting and demographic bias; parsimony, exact-logistic checks, and bootstrap intervals were used to temper optimism, and external validation with subgroup fairness testing is warranted.

In summary, tumor biology—particularly LVI—provides a more stable prognostic signal than TTS. A-TTS and B-TTS are complementary process measures; centers may monitor both in parallel, document brief reason-coded extensions when targets are exceeded, and trigger concise case reviews aligned with national frameworks (e.g., 31- and 62-day pathways) [1,21,27,28]. Future work should standardize delay categories, apply competing-risks methods for cause-specific outcomes, and evaluate TTI alongside A-TTS and B-TTS [33,34].

## 5. Conclusions

LVI appears to provide a more stable prognostic signal than TTS in this cohort. A-TTS ≤ 24 days was associated with recurrence and an early, attenuating mortality hazard, whereas B-TTS showed no association—supporting these intervals as complementary process measures rather than biological surrogates. In practice, parallel monitoring and brief reviews of threshold breaches may support quality while avoiding unnecessary haste, recognizing that some extensions may reflect necessary evaluation or planning. Because urgency perceptions can be shaped by patient anxiety and medicolegal context, signals should be interpreted cautiously and validated prospectively. Parallel reporting of these intervals as service-quality indicators may support governance and patient safety frameworks.

## Figures and Tables

**Table 1:** Baseline clinicopathologic characteristics (*n* = 167).

Variable	Category	*n* (%)
Age category	<40 years	32 (19.2)
	≥40 years	135 (80.8)
Family history of breast cancer	No	131 (78.4)
	Yes	36 (21.6)
MMG performed	No	33 (19.8)
	Yes	134 (80.2)
Breast MRI performed	No	94 (56.3)
	Yes	73 (43.7)
Histology (grouped)	NST and unfavorable subtypes	137 (82.0)
	ILC (classic)	17 (10.2)
	Favorable subtypes	13 (7.8)
Pathologic tumor size (TNM)	≤20 mm	44 (26.3)
	>20 to ≤50 mm	101 (60.5)
	>50 mm	22 (13.2)
Histologic grade	Grade I	10 (6.0)
	Grade II	74 (44.3)
	Grade III	83 (49.7)
DCIS component	No (absent)	53 (31.7)
	Yes (present)	114 (68.3)
Multifocality/multicentricity	No	132 (79.0)
	Yes	35 (21.0)
Lymphovascular invasion	Absent	73 (43.7)
	Present	94 (56.3)
Perineural invasion	Absent	105 (62.9)
	Present	62 (37.1)
N category (TNM)	N0	69 (41.3)
	N1 (1–3 nodes)	57 (34.1)
	N2 (4–9 nodes)	27 (16.2)
	N3 (≥10 nodes)	14 (8.4)
Pathologic stage (anatomic)	Stage I	41 (24.6)
	Stage II	106 (63.4)
	Stage III	20 (12.0)
Intrinsic subtype (IHC-based)	Luminal A	104 (62.3)
	Luminal B	35 (21.0)
	HER2-enriched (non-luminal)	18 (10.8)
	TNBC	10 (6.0)
HER2 status	HER2 negative	138 (82.6)
	HER2 positive	29 (17.4)
Surgery type	BCS + SLNB	14 (8.4)
	BCS + ALND	11 (6.6)
	Mastectomy + SLNB	55 (32.9)
	Mastectomy + ALND	87 (52.1)
Adjuvant chemotherapy	No	16 (9.6)
	Yes	151 (90.4)
Adjuvant radiotherapy	No	56 (33.5)
	Yes	111 (66.5)
Adjuvant endocrine therapy	No	39 (23.4)
	Yes	128 (76.6)
Recurrence during follow-up	No	149 (89.2)
	Yes	18 (10.8)
Death during follow-up	No	144 (86.2)
	Yes	23 (13.8)
Timing thresholds (A-TTS)	A-TTS ≤ 24 days: Yes	41 (24.6)
	A-TTS ≤ 24 days: No	126 (75.4)
	A-TTS ≤ 30 days: Yes	67 (40.1)
	A-TTS ≤ 30 days: No	100 (59.9)
Timing thresholds (B-TTS)	B-TTS ≤ 24 days: Yes	123 (73.7)
	B-TTS ≤ 24 days: No	44 (26.3)
	B-TTS ≤ 30 days: Yes	134 (80.2)
	B-TTS ≤ 30 days: No	33 (19.8)

Notes. Data are shown as *n* (%). Histology groups: NST and unfavorable (NST/mixed/micropapillary/metaplastic/pleomorphic ILC/other rare), ILC (classic), favorable subtypes (mucinous/papillary/cribriform/tubular). TNM categories reflect pathologic staging. Surgical procedures are recorded as BCS/mastectomy with SLNB and/or ALND. Abbreviations: MMG, mammography; MRI, magnetic resonance imaging; NST, no special type; ILC (invasive lobular carcinoma); unfavorable subtypes: mixed, micropapillary subtypes, metaplastic carcinoma, pleomorphic ILC; favorable subtypes: mucinous, papillary, cribriform, tubular subtypes; TNM, tumor node metastasis; DCIS, ductal carcinoma in situ; IHC, immunohistochemistry; HER2, human epidermal growth factor receptor 2; TNBC, triple-negative breast cancer; BCS, breast-conserving surgery; SLNB, sentinel lymph node biopsy; ALND, axillary lymph node dissection; A-TTS, admission-to-surgery; B-TTS, biopsy-result-to-surgery.

**Table 2:** Continuous variables by recurrence status (*n* = 167).

Variable	No Recurrence (*n* = 149)	Recurrence (*n* = 18)	*p*-Value
Age (years)	50.11 ± 11.02	47.06 ± 8.93	0.196
Tumor size at biopsy (mm)	20.0 (14.0–25.0)	30.0 (20.0–40.0)	**0.001**
Tumor size at pathology (mm)	25.0 (18.0–37.0)	45.0 (29.3–52.8)	**0.001**
Ki-67 (%)	20.0 (10.0–30.0)	15.0 (10.0–32.5)	0.758
A-TTS (days)	35.0 (26.0–52.0)	24.0 (17.5–41.3)	**0.017**
B-TTS (days)	16.0 (9.5–26.5)	11.0 (4.8–24.0)	0.168
A–B (days)	16.0 (10.0–26.5)	11.0 (9.0–14.0)	0.017
Surgery-to-adjuvant (days)	42.0 (33.0–55.0)	53.0 (33.5–62.0)	0.274

Notes: Age is mean ± SD with *p* from the *t*-test; all other variables are median (IQR, 25th–75th percentile) with *p* from the two-sided Mann–Whitney U test (α = 0.05). Bold *p*-values indicate statistical significance at the 0.05 level. Tumor sizes are in millimeters; intervals are in days. A–B denotes the within-patient difference (A-TTS minus B-TTS). “Surgery-to-adjuvant” is the interval from surgery date to first adjuvant treatment (chemotherapy, radiotherapy, or endocrine therapy). Abbreviations: A-TTS, admission-to-surgery; B-TTS, biopsy-result-to-surgery; A–B = within-patient difference (A-TTS minus B-TTS).

**Table 3:** Logistic regression for recurrence (primary parsimonious model).

Variable	Odds Ratio	95% CI	*p*-Value
A-TTS ≤ 24 days (yes vs. no)	3.16	1.13–8.82	**0.028**
Node-positive disease (N1–3 vs. N0)	1.98	0.39–10.09	0.412
LVI (yes vs. no)	2.52	0.50–12.79	0.265

Notes: Model includes A-TTS ≤ 24 days, node-positive disease (N1–3), and LVI (*n* = 167; events = 18). Odds ratios are Wald estimates with 95% CIs. Model discrimination AUC = 0.733 (95% CI, 0.620–0.847); Hosmer–Lemeshow goodness-of-fit *p* ≈ 0.997. Covariates were prespecified a priori to respect events-per-parameter. Bold *p*-values indicate statistical significance at the 0.05 level. Abbreviations: A-TTS, admission-to-surgery; LVI, lymphovascular invasion; AUC, Area Under the Curve.

**Table 4:** Overall survival models: Panel A (Cox proportional hazards) and Panel B (log–time interaction centered at 24 months).

**Panel A. Cox Proportional Hazards (Baseline Model)**		
**Variable**	**HR (95% CI)**	***p*-Value**
A-TTS ≤ 24 days (yes vs. no)	1.73 (0.75–4.01)	0.201
Node-positive disease (N1–3 vs. N0)	1.23 (0.36–4.21)	0.737
LVI (yes vs. no)	3.93 (1.03–14.99)	**0.045**
**Panel B. Extended Cox Model with Log–Time Interaction ln(time/24); the Main Effect Corresponds to the HR at 24 Months**		
**Effect**	**HR (95% CI)**	
A-TTS ≤ 24 days (HR at 24 months)	22.83 (6.44–80.98)	
A-TTS ≤ 24 × log(time/24)	0.06 (0.02–0.21)	

Notes: Panel A shows proportional-hazards (PHs) estimates (time in months). Panel B is an extended Cox model with a log–time interaction ln(time/24); the main effect corresponds to the HR at 24 months. In Panel B, both effects had *p* < 0.001 (Wald tests). Bold *p*-values indicate statistical significance at the 0.05 level. Abbreviations: A-TTS, admission-to-surgery; TNM, tumor node metastasis; LVI, lymphovascular invasion; HR, hazard ratio; CI, confidence interval; OS, overall survival.

## Data Availability

De-identified data are available from the corresponding author upon reasonable request and institutional approvals. Upon request, analysis materials (sufficient to reproduce the main tables and figures) will be provided.

## Supplementary Materials

The following supporting information can be downloaded at https://www.mdpi.com/article/10.3390/healthcare13233010/s1: Supplementary Figure S1: ROC curve for the parsimonious logistic model (area under the curve and 95% confidence interval). Supplementary Figure S2: Calibration of the parsimonious logistic model: apparent vs. bias-corrected curves and Hosmer–Lemeshow *p*-value. Supplementary Figure S3: Distributions of admission-to-surgery (A-TTS), biopsy-result-to-surgery (B-TTS), and within-patient differences (A–B), with medians and interquartile ranges. Supplementary Figure S4: Forest plot of adjusted odds ratios for recurrence (95% confidence intervals; estimates correspond to Table 3). Supplementary Figure S5: Overall survival by B-TTS ≤24 days versus >24 days (Kaplan–Meier). Supplementary Table S1: Pairwise associations among candidate predictors. Supplementary Table S2: Collinearity diagnostics (variance inflation factors). Supplementary Table S3: Agreement between timing definitions (A-TTS vs. B-TTS) at 24- and 30-day thresholds. Supplementary Table S4: Confusion matrices for the parsimonious logistic regression model at two probability thresholds. Supplementary Table S5: Paired comparison of logistic regression versus decision tree. Supplementary Table S6: Comparison of clinicopathologic variables between patients with and without recurrence.

## Abbreviations

A-TTS, admission-to-surgery; AJCC, American Joint Committee on Cancer; B-TTS, biopsy-result-to-surgery; AUC, area under the curve; ALND, axillary lymph node dissection; BCS, breast-conserving surgery; CI, confidence interval; DCIS, ductal carcinoma in situ; EPV, events-per-parameter; ER, estrogen receptor; PR, progesterone receptor; HER2, human epidermal growth factor receptor 2; HR, hazard ratio; IHC, immunohistochemistry; ILC, invasive lobular carcinoma; IQR, interquartile range; LVI, lymphovascular invasion; MDT, multidisciplinary team; MMG, mammography; MRI, magnetic resonance imaging; NAST, neoadjuvant systemic therapy; NPV, negative predictive value; NST, no special type; OLS, ordinary least squares; OR, odds ratio; OS, overall survival; PH, proportional hazards; PNI, perineural invasion; PPV, positive predictive value; PS, propensity score; ROC, receiver-operating characteristic; RT, radiotherapy; SD, standard deviation; SLNB, sentinel lymph node biopsy; STROBE, Strengthening the Reporting of Observational Studies in Epidemiology; TNBC, triple-negative breast cancer; TNM, tumor–node–metastasis; TTI, time-to-treatment initiation; TTS, time-to-surgery; UK, United Kingdom; US, ultrasonography; VIF, variance inflation factors.
