# Supplementary material for: Measuring Surgical Waiting Times in Breast Cancer: Admission to Surgery Versus Biopsy Result to Surgery
Source: Healthcare (Basel). 2025 Nov 21;13(23):3010. doi: 10.3390/healthcare13233010 (PMC12692320; doi:10.3390/healthcare13233010)
Supplement: Supplementary file 1 [file healthcare-13-03010-s001.zip › Supplementary Figures.pdf]

Supplementary Figures

All point estimates are shown or referenced with 95% CIs.

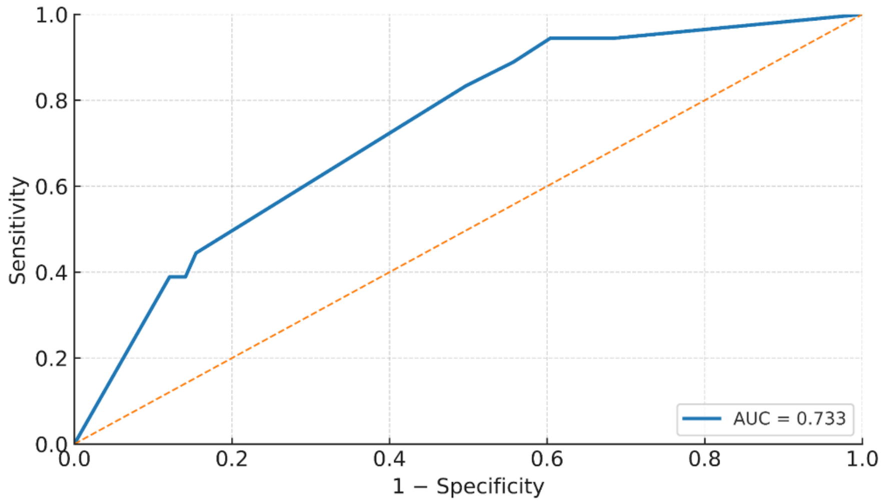

Supplementary Figure S1. ROC curve for the parsimonious logistic model (area under the curve and 95% confidence interval).

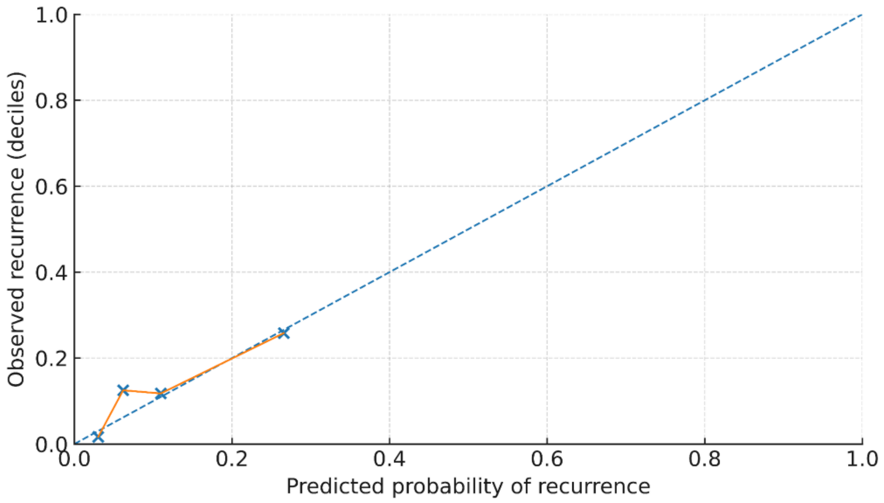

Supplementary Figure S2. Calibration of the parsimonious logistic model: apparent vs. bias-corrected curves and Hosmer–Lemeshow p-value.

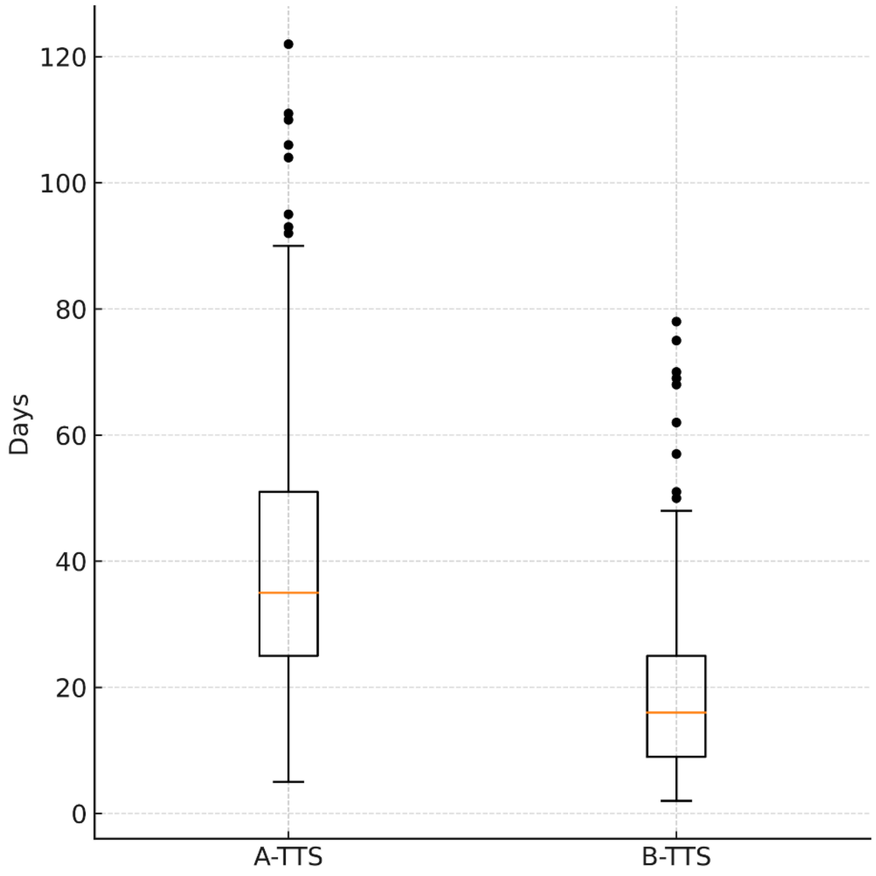

Supplementary Figure S3. Distributions of A-TTS and biopsy-result-to-surgery (B-TTS) and within-patient differences (A–B) with medians and interquartile ranges.

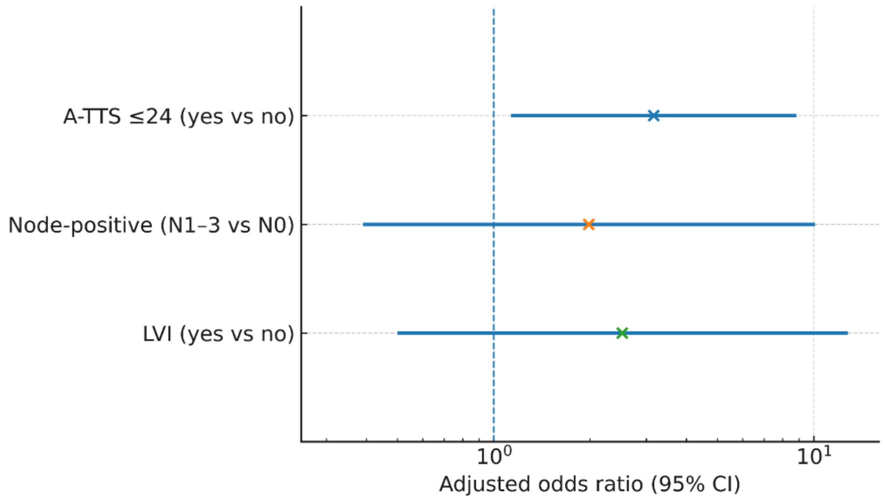

Supplementary Figure S4. Forest plot of adjusted odds ratios for recurrence (95% confidence intervals; estimates correspond to Table 3).

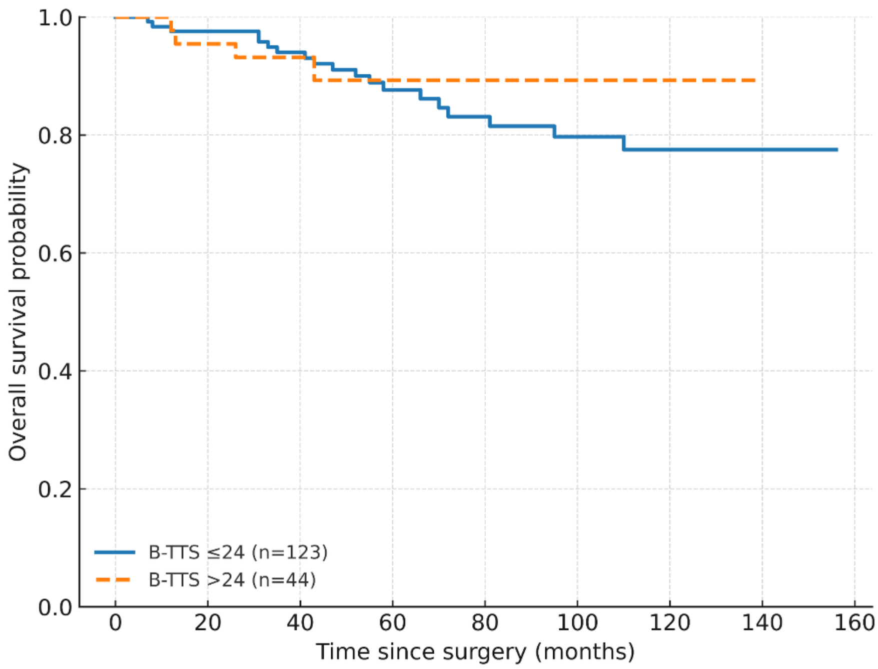

Supplementary Figure S5. Overall survival by B-TTS ≤24 days versus >24 days (Kaplan–Meier).
