# Supplementary material for: Measuring Surgical Waiting Times in Breast Cancer: Admission to Surgery Versus Biopsy Result to Surgery
Source: Healthcare (Basel). 2025 Nov 21;13(23):3010. doi: 10.3390/healthcare13233010 (PMC12692320; doi:10.3390/healthcare13233010)
Supplement: Supplementary file 1 [file healthcare-13-03010-s001.zip › Supplementary Tables.pdf]

Supplementary Tables

Supplementary Table S1. Pairwise associations among candidate predictors (n=167).

Variable 1	Variable 2	Association	Estimate	p-value
Pathologic stage I–II vs III (coding A)	Pathologic stage I–II vs III (coding B)	Cramér's V	1.000	**<0.001**
A-TTS ≤24 days (0/1) (coding A)	A-TTS ≤24 days (0/1) (coding B)	Cramér's V	1.000	**<0.001**
Pathologic stage I–II vs III	Pathologic tumor size ≤50 vs >50 mm	Cramér's V	0.971	**<0.001**
Pathologic tumor size ≤50 vs >50 mm	Tumor size category 0–50 vs >50 mm	Cramér's V	0.919	**<0.001**
Lymphovascular invasion (0/1)	Axillary surgery: SLNB vs ALND	Cramér's V	0.556	**<0.001**
Clinical nodal status N2–3 (0/1)	Axillary surgery: SLNB vs ALND	Cramér's V	0.474	**<0.001**
Nottingham grade I–II vs III	Intrinsic subtype (IHC) (collapsed 1–3)	Cramér's V	0.339	**<0.001**
A-TTS ≤24 days (0/1)	B-TTS ≤24 days (0/1)	Cramér's V	0.333	**<0.001**
Lymphovascular invasion (0/1)	Pathologic tumor size ≤50 vs >50 mm	Cramér's V	0.307	**<0.001**
B-TTS (days)	A-TTS (days)	Spearman ρ	0.650	**<0.001**

Notes: *Association type: Cramér's V for categorical–categorical, Spearman ρ for continuous–continuous, point-biserial r for continuous–binary. Very high values (≈1.00) indicate deterministic/near-deterministic overlap. P values are unadjusted; n = number of complete pairs.* ***Abbreviations:*** *A-TTS, admission-to-surgery; B-TTS, biopsy-result-to-surgery; ALND,*

axillary lymph node dissection; SLNB, sentinel lymph node biopsy; intrinsic subtype 'IHC-based', immunohistochemistry.

Supplementary Table S2. Collinearity diagnostics (variance inflation factors, VIFs) (n=167).

Predictor	VIFs
A-TTS ≤24 days (0/1)	∞
A-TTS ≤24 days (0/1)	∞
Pathologic stage I–II vs III	∞
Pathologic stage I–II vs III	∞
Pathologic tumor size ≤50 vs >50 mm	24.522
Tumor size category 0–50 vs >50 mm	6.820
Lymphovascular invasion (0/1)	1.275
Clinical nodal status N2–3 (0/1)	1.147
Nottingham grade I–II vs III	1.094
Breast surgery: BCS vs mastectomy	1.077
Age ≤50 vs >50 years	1.056

Notes: *VIF > 10 suggests substantial collinearity; VIFs ≈ ∞ denotes perfect/near-perfect collinearity. Practical rule: keep only one from each collinear pair, e.g., {Pathologic stage I–II vs III, Pathologic stage I–II vs III (alternative coding)}; {A-TTS ≤24 days, A-TTS ≤24 days (alternative coding)}; {Pathologic tumor size ≤50 vs >50 mm, Tumor size category 0–50 vs >50 mm}.* ***Abbreviations:*** *A-TTS, admission-to-surgery; BCS, breast-conserving surgery; VIFs, variance inflation factors.*

Supplementary Table S3. Agreement between timing definitions (A-TTS vs B-TTS) (n=167).

**Panel A — Threshold ≤24 days**		
	**B-TTS ≤ threshold**	**B-TTS > threshold**
A-TTS ≤ threshold	41	0
A-TTS > threshold	82	44
**Overall agreement**	50.9% (85/167)	
**Cohen's κ (95% CI)**	0.21 (0.14–0.29)	
**Positive percent agreement**	50.0%	
**Negative percent agreement**	51.8%	
**McNemar test (discordance)**	χ^2=80.0; **p<0.001**	
**Panel B — Threshold ≤30 days**		
	**B-TTS ≤ threshold**	**B-TTS > threshold**
A-TTS ≤ threshold	67	0
A-TTS > threshold	67	33
**Overall agreement**	59.9% (100/167)	
**Cohen's κ (95% CI)**	0.28 (0.19–0.38)	
**Positive percent agreement**	66.7%	
**Negative percent agreement**	49.6%	
**McNemar test (discordance)**	χ^2=65.0; **p<0.001**	

Notes: *Cohen's κ 95% CIs were estimated by bias-corrected and accelerated (BCa) bootstrap, 5,000 resamples; the exact McNemar test was used for marginal homogeneity.* ***Abbreviations:*** *A-TTS, admission-to-surgery; B-TTS, biopsy-result-to-surgery.*

Supplementary Table S4. Confusion matrices for the parsimonious logistic regression model at two thresholds (n=167).

**Panel A — Youden J cut-point**		
**Probability threshold:** 0.062 (derived from ROC on model-predicted probability)		
**Confusion matrix (n=167)**		
	**Predicted positive**	**Predicted negative**
**Actual positive**	TP = 17	FN = 1
**Actual negative**	FP = 90	TN = 59
**Operating characteristics**		
Sensitivity	94.4%	
Specificity	39.6%	
PPV	15.9%	
NPV	98.3%	
Accuracy	45.5%	
F1-score	0.272	
Predicted positives	107 (64.1%)	
**Panel B — Prevalence-matched threshold**		
**Probability threshold:** 0.292 (predicted positives ≈ 15.0%; 25/167)		
**Confusion matrix (n=167)**		
	**Predicted positive**	**Predicted negative**
**Actual positive**	TP = 7	FN = 11
**Actual negative**	FP = 18	TN = 131
**Operating characteristics**		
Sensitivity	38.9%	

Specificity	87.9%
PPV	28.0%
NPV	92.3%
Accuracy	82.6%
F1-score	0.326
Predicted positives	25 (15.0%)

Notes: *Predictors: A-TTS ≤24 days, node-positive disease (N1–3), and LVI. Metrics were derived from predictions of the parsimonious logistic regression model in the full cohort (n=167). Formulas: Sensitivity = TP/(TP+FN); Specificity = TN/(TN+FP); PPV = TP/(TP+FP); NPV = TN/(TN+FN); Accuracy = (TP+TN)/n; F1 = 2·(PPV·Sensitivity)/(PPV+Sensitivity). Because multiple cases share identical predicted probabilities, the prevalence-matched threshold yields ≈15% predicted positives (25/167), the closest feasible match to the observed prevalence (10.8%).* ***Abbreviations:*** *A-TTS, admission-to-surgery; LVI, lymphovascular invasion; PPV, positive predictive value; NPV, negative predictive value; TP, true positive; FN, false negative; FP, false positive; TN, true negative.*

Supplementary Table S5. Paired comparison of logistic regression (LR) versus decision tree (DT) (n=163).

**Panel A — AUC comparison (paired DeLong test)**			
**Metric**	**Estimate**	**95% CI**	**p-value**
AUC (LR)	0.729	—	—
AUC (DT)	0.753	—	—
ΔAUC (LR−DT)	−0.024	−0.096 to 0.047	0.507

**Panel B — McNemar tests at two thresholds**			
**Threshold definition**	**Discordant (LR✓/DT✗ ; LR✗/DT✓)**	**Test**	**p-value**
Model-specific Youden-J	1 ; 12	Exact McNemar	**0.003**
Prevalence-matched	8 ; 13	Exact McNemar	0.383

**Panel C — Bootstrap paired differences in operating metrics (LR−DT)**			
**Threshold**	**Metric**	**Median Δ**	**95% CI**
Youden-J	Accuracy	−0.066	−0.108 to −0.024
Youden-J	F1-score	−0.015	−0.043 to 0.026
Youden-J	PPV	−0.012	−0.031 to 0.011
Youden-J	NPV	0.010	−0.008 to 0.046
Prevalence-matched	Accuracy	−0.030	−0.084 to 0.024
Prevalence-matched	F1-score	−0.011	−0.058 to 0.033
Prevalence-matched	PPV	−0.008	−0.036 to 0.019
Prevalence-matched	NPV	−0.001	−0.042 to 0.036

Notes. *Youden-J cut-points were derived from ROC coordinate tables separately for each model. The prevalence-matched threshold was set to the observed outcome prevalence. McNemar discordant pairs are reported as LR✓/DT✗ ; LR✗/DT✓; p-values are exact and two-sided. Bootstrap resampling was performed at the patient level (B=2,000; with replacement); medians and percentile 95% CIs are shown for paired differences (LR−DT). All tests are two-sided (α=0.05); inferences should be interpreted with caution given the small number of events.* ***Abbreviations:*** *LR, logistic regression; DT, decision tree; AUC, area under the ROC curve; CI, confidence interval; PPV, positive predictive value; NPV, negative predictive value.*

Supplementary Table S6. Comparison of clinicopathological variables between patients with and without recurrence (n=167).

Variable	No recurrence n (%)	Recurrence n (%)	p-value
Age category			1.000
<40 years	29 (19.5)	3 (16.7)	
≥40 years	120 (80.5)	15 (83.3)	
Family history of breast cancer			0.545
No	118 (79.2)	13 (72.2)	
Yes	31 (20.8)	5 (27.8)	
Histology			0.700
NST and unfavorable subtypes	122 (81.9)	15 (83.3)	
ILC (classic)	16 (10.7)	1 (5.6)	
Favorable subtypes	11 (7.4)	2 (11.1)	
Pathologic tumor size (TNM)			**0.007**
≤50 mm	133 (89.3)	12 (66.7)	
>50 mm	16 (10.7)	6 (33.3)	
Histologic grade			0.127
Grade I–II	78 (52.3)	6 (33.3)	
Grade III	71 (47.7)	12 (66.7)	
DCIS component			0.878
No (absent)	47 (31.5)	6 (33.3)	
Yes (present)	102 (68.5)	12 (66.7)	
Multifocality/multicentricity			0.217
No	120 (80.5)	12 (66.7)	

Yes	29 (19.5)	6 (33.3)	
Lymphovascular invasion			**0.014**
Absent	70 (47.0)	3 (16.7)	
Present	79 (53.0)	15 (83.3)	
Perineural invasion			**0.026**
Absent	98 (65.8)	7 (38.9)	
Present	51 (34.2)	11 (61.1)	
Nodal status (TNM)			**0.003**
N0	66 (44.3)	3 (16.7)	
N1 (1–3 nodes)	52 (34.9)	5 (27.8)	
N2 (4–9 nodes)	22 (14.8)	5 (27.8)	
N3 (≥10 nodes)	9 (6.0)	5 (27.8)	
Pathologic stage (anatomic)			**0.045**
Stage I–II	134 (89.9)	13 (72.2)	
Stage III	15 (10.1)	5 (27.8)	
Intrinsic subtype (IHC-based)			0.109
Luminal A-B	125 (83.9)	14 (77.8)	
HER2-enriched (non-luminal)	17 (11.4)	1 (5.6)	
TNBC	7 (4.7)	3 (16.7)	
HER2 status			0.742
Negative	122 (81.9)	16 (88.9)	
Positive	27 (18.1)	2 (11.1)	
Breast surgery type			0.236
Breast conserving surgery	24 (16.1)	1 (5.6)	
Mastectomy-based surgery	125 (83.9)	17 (94.4)	

Axillary surgery type			0.082
SLNB	65 (43.6)	4 (22.2)	
ALND (including completion)	84 (56.4)	14 (77.8)	
Adjuvant radiotherapy			**0.008**
No	55 (36.9)	1 (5.6)	
Yes	94 (63.1)	17 (94.4)	
Adjuvant chemotherapy			1.000
No	15 (10.1)	1 (5.6)	
Yes	134 (89.9)	17 (94.4)	
Adjuvant endocrine therapy			0.137
No	32 (21.5)	7 (38.9)	
Yes	117 (78.5)	11 (61.1)	
A-TTS threshold			**0.017**
>24 days	117 (78.5)	9 (50.0)	
≤24 days	32 (21.5)	9 (50.0)	
B-TTS threshold			0.407
>24 days	41 (27.5)	3 (16.7)	
≤24 days	108 (72.5)	15 (83.3)	
Death during follow-up			**<0.001**
Alive	137 (91.9)	7 (38.9)	
Death	12 (8.1)	11 (61.1)	

Notes: *Values are given as n (%). p-values are from Chi-square or Fisher's exact test for categorical variables. Bold p-values indicate statistical significance at the 0.05 level. Percentages are column-wise; totals may not equal 100 because of rounding. Thresholds were set at ≤24 and ≤30 days for A-TTS and B-TTS. N category refers to pathologic TNM nodal*

status. Postoperative therapies reflect indication and disease burden; unadjusted associations with recurrence may be confounded by indication. ***Abbreviations:*** *NST, no special type; ILC, invasive lobular carcinoma; unfavorable subtypes: mixed, micropapillary subtypes, metaplastic carcinoma, pleomorphic ILC; favorable subtypes: mucinous, papillary, cribriform, tubular subtypes; TNM, tumor node metastasis; DCIS, ductal carcinoma in situ; IHC, immunohistochemistry; HER2, human epidermal growth factor receptor 2; TNBC, triple-negative breast cancer; BCS, breast-conserving surgery; SLNB, sentinel lymph node biopsy; ALND, axillary lymph node dissection; A-TTS, admission-to-surgery; B-TTS, biopsy-result-to-surgery.*
